# Polyoxygenated Cholesterol Ester Hydroperoxide Activates TLR4 and SYK Dependent Signaling in Macrophages

**DOI:** 10.1371/journal.pone.0083145

**Published:** 2013-12-23

**Authors:** Soo-Ho Choi, Huiyong Yin, Amir Ravandi, Aaron Armando, Darren Dumlao, Jungsu Kim, Felicidad Almazan, Angela M. Taylor, Coleen A. McNamara, Sotirios Tsimikas, Edward A. Dennis, Joseph L. Witztum, Yury I. Miller

**Affiliations:** 1 Department of Medicine, University of California San Diego, La Jolla, California, United States of America; 2 Key Laboratory of Food Safety Research, Institute for Nutritional Sciences, Shanghai Institutes for Biological Sciences, Chinese Academy of Sciences, Shanghai, China; 3 Key Laboratory of Food Safety Risk Assessment, Ministry of Health, Beijing, China; 4 School of Life Science and Technology, Shanghai Tech University, Shanghai, China; 5 Institute of Cardiovascular Sciences, University of Manitoba, Winnipeg, Manitoba, Canada; 6 Department of Pharmacology, University of California San Diego, La Jolla, California, United States of America; 7 Department of Chemistry and Biochemistry, University of California San Diego, La Jolla, California, United States of America; 8 Cardiovascular Research Center, Department of Medicine, University of Virginia, Charlottesville, Virginia, United States of America; University of California Merced, United States of America

## Abstract

Oxidation of low-density lipoprotein (LDL) is one of the major causative mechanisms in the development of atherosclerosis. In previous studies, we showed that minimally oxidized LDL (mmLDL) induced inflammatory responses in macrophages, macropinocytosis and intracellular lipid accumulation and that oxidized cholesterol esters (OxCEs) were biologically active components of mmLDL. Here we identified a specific OxCE molecule responsible for the biological activity of mmLDL and characterized signaling pathways in macrophages in response to this OxCE. Using liquid chromatography – tandem mass spectrometry and biological assays, we identified an oxidized cholesteryl arachidonate with bicyclic endoperoxide and hydroperoxide groups (BEP-CE) as a specific OxCE that activates macrophages in a TLR4/MD-2-dependent manner. BEP-CE induced TLR4/MD-2 binding and TLR4 dimerization, phosphorylation of SYK, ERK1/2, JNK and c-Jun, cell spreading and uptake of dextran and native LDL by macrophages. The enhanced macropinocytosis resulted in intracellular lipid accumulation and macrophage foam cell formation. Bone marrow-derived macrophages isolated from TLR4 and SYK knockout mice did not respond to BEP-CE. The presence of BEP-CE was demonstrated in human plasma and in the human plaque material captured in distal protection devices during percutaneous intervention. Our results suggest that BEP-CE is an endogenous ligand that activates the TLR4/SYK signaling pathway. Because BEP-CE is present in human plasma and human atherosclerotic lesions, BEP-CE-induced and TLR4/SYK-mediated macrophage responses may contribute to chronic inflammation in human atherosclerosis.

## Introduction

Oxidized low density lipoprotein (LDL) is considered a major pathogenic factor in the development of atherosclerosis [1], [2]. Many studies documented proinflammatory and atherogenic effects of OxLDL produced by *in vitro* oxidation of native LDL [3], [4]. Importantly, even though LDL in these studies was oxidized *in vitro*, a number of biologically active molecules identified in *in vitro* oxidized LDL have been also found in plasma and/or atherosclerotic lesions of humans and experimental animals [3], [5]–[8]. These findings suggest that the mechanisms of OxLDL-induced activation of macrophages, endothelial cells and vascular smooth muscle cells are relevant to the initiation and progression of atherosclerosis.

Oxidized phospholipids containing a phosphocholine headgroup (OxPL) have been the focus of many recent studies, revealing molecular structures of OxPLs, their cellular and soluble receptors, and characteristic inflammatory and atherogenic responses to these OxPLs [4], [6], [9], [10]. A stable OxPL mimic has been synthesized to facilitate further biological studies [11]. Less is known about biological activity of other major components of OxLDL, such as oxidized cholesterol esters (OxCEs). We and others have documented accumulation of OxCE in the lesions of *Apoe^−/−^* and *Ldlr^−/−^* mice fed a high-fat diet (HFD), zebrafish fed a high-cholesterol diet, as well as in human atherosclerotic tissue [8], [12]–[16]. OxCE stimulates endothelial cells to bind monocytes via endothelial connecting segment-1 [17]. OxCE is the most abundant class of oxidized lipids in minimally oxidized LDL (mmLDL) [14], [18]. Our previous studies have demonstrated robust macrophage responses to mmLDL, including membrane ruffling, cell spreading, macropinocytosis, lipoprotein uptake, rescue of macrophage foam cells from apoptosis, ROS generation, inflammatory signaling and cytokine secretion, as well as cooperative inflammatory activation with low dose LPS [19]–[25]. The majority of these effects in macrophages were mediated by TLR4/MD-2- and SYK-dependent signaling [19], [20], [22], [23], [25].

In the current study, using liquid chromatography – tandem mass spectrometry (LC-MS/MS), we identified a specific biologically active OxCE, an oxidized, polyoxygenated cholesteryl arachidonate with bicyclic endoperoxide and hydroperoxide groups (BEP-CE). We documented the presence of BEP-CE in human plasma and in human atherosclerotic lesions. BEP-CE activated macrophages via TLR4/MD-2 and SYK to secrete CXCL2 (MIP-2) and accumulate lipid. Our findings suggest that BEP-CE is an endogenously generated agonist of TLR4 and as such it may contribute to development of atherosclerosis.

## Materials and Methods

### Ethics Statement

All animal experiments were performed according to the NIH guidelines and were approved by the Animal Subjects Committee of the UC San Diego (protocol S04155). Human plasma, used for LDL isolation, was obtained from normal volunteers who provided written informed consent according to a protocol approved by the UC San Diego Human Research Protection Program (project #71402). The collection of human blood samples from participants who provided written informed consent was approved by the Institutional Review Board for Health Sciences Research at the University of Virginia (project #14620). The collection of material from distal protection devices, with written informed consent, was approved by the UC San Diego Human Research Subjects Protection Program (project #90696).

### Human Plasma and Atherosclerotic Plaque Material

Twelve human blood samples were collected at the University of Virginia Cardiac Catheterization laboratory, with the approval by the Institutional Review Board for Health Sciences Research at the University of Virginia. Blood was drawn into tubes containing EDTA. Immediately upon plasma separation, it was supplemented with 20 µM butylated hydroxytoluene (BHT) to prevent *ex vivo* oxidation and frozen at −80°C.

Nine distal protection devices were obtained from patients undergoing clinically indicated coronary and peripheral procedures, which included stent placement in all cases, at the University of California, San Diego Sulpizio Cardiovascular Center. Among the patients: 3 had saphenous vein graft intervention; 2 had superficial femoral artery intervention because of claudication; 1 had renal artery intervention because of stenosis and uncontrolled hypertension on maximal medical therapy; and 3 had intervention because of >80% stenosis without prior stroke or transient ischemic attack. The collection of materials was approved by the UC San Diego Human Research Subjects Protection Program.

Filters from distal protection devices were immediately placed in ice-cold PBS containing 4 µM EDTA and 20 µM BHT to arrest any *ex vivo* oxidation. The filters were then extracted by the Folch method [26]. Briefly, 500 µl of filter material homogenate in a glass tube was vigorously vortexed with 1.25 ml of ice-cold chloroform/methanol (1∶2) containing CE 17∶0 as an internal standard. Phase separation was achieved with addition of 1.85 ml chloroform. After centrifugation, the lower organic phase was collected, dried under argon and reconstituted in LC mobile phase prior to injection. A similar protocol, with internal standard, was used for lipid extraction from 200 µl of human plasma.

### Animals

Myeloid cell specific SYK knockdown mice were generated by breeding *Syk^flox/flox^* mice with *LysM-cre* mice as described [25]. We further refer to *Syk^flox/flox^/LysM-cre(+)* mice as *Syk^−/−^*, and to their littermate, *Syk^flox/flox^LysM-cre(−)* mice as wild type (WT). *Ldlr^−/−/^Tlr4^−/−^* double knockout mice [27] were kindly provided by Dr. Peter Tobias (Scripps Research Institute), and age and gender matched *Ldlr^−/−^* mice were purchased from the Jackson Laboratory and used as a control. We use *Ldlr^−/−/^Tlr4^−/−^* double knockout mice because, under our housing conditions, they have higher survival and fertility rates compared to *Tlr4^−/−^* mice. Mice were housed in a barrier facility with a 12-hour light/12-hour dark cycle, and fed normal mouse chow containing 4.5% fat (Harlan Teklad). All animal experiments were approved by the UC San Diego Institutional Animal Care and Use Committee.

### Cell Culture

Murine macrophage-like J774A.1 cells (ATCC) were cultured in DMEM supplemented with 10% FBS and 50 µg/ml gentamicin (Calbiochem). Bone marrow-derived macrophages (BMDM) were obtained by incubating bone marrow cells isolated from tibias and femurs of WT, *Syk^−/−^*, *Ldlr^−/−^* and *Ldlr^−/−/^Tlr4^−/−^* mice with macrophage colony stimulating factor (L929 conditioned medium) following the published protocols [25], [28]. Ba/F3 cells stably expressing TLR4-gfp and TLR4-flag [29], [30] were cultured in RPMI1640 medium (Invitrogen) supplemented with murine interleukin-3 [31], 10% FBS and 50 µg/ml gentamicin. To generate HEK293 cells stably expressing both MD-2-myc-his and the extracellular domain of TLR4-flag-his, cells were transfected with each expression plasmids using GenJet In Vitro DNA transfection reagent (SignaGen Laboratories). Single colonies were selected in the presence of 200 µg/ml Zeocin (Invitrogen) for MD2-myc-his and 500 µg/ml G418 (Omega Scientific) for TLR4-flag-his selection. Positive clones were selected by immunoblot analysis using anti-myc and anti-flag antibodies. Cells were cultured in DMEM supplemented with 10% FBS, 50 µg/ml gentamicin, 500 µg/ml G418, and 200 µg/ml Zeocin to maintain selection.

### LDL Isolation, Modification and Lipid Extraction

Human plasma, used for LDL isolation, was obtained from normal volunteers according to a protocol approved by the UC San Diego Human Research Protection Program. Native LDL (density = 1.019–1.063 g/ml) was isolated by sequential ultracentrifugation [32]. Endotoxin levels were determined using a LAL assay (Lonza), and preparations with the endotoxin levels below 0.025 EU/mg protein were used in experiments. To produce mmLDL, 50 µg/ml of LDL was incubated in serum-free DMEM for 18 hours with murine fibroblast cells overexpressing human 15-lipoxygenase (15LO), as reported in detail [19], [22]. Lipids were isolated from mmLDL using a hexane/methanol extraction procedure described earlier [14], which ensures high yield of CE extraction.

### Oxidation of Cholesteryl Arachidonate

Arachidonic acid cholesteryl ester (AA-CE; purchased from Nu-Check) was reconstituted in hexane at 2.5 mg/ml and kept at −80°C. Both enzymatic and free radical oxidation reaction protocols were used. For enzymatic oxidation, 50 µg of AA-CE was incubated with 24,000 units of soybean 15LO (Cayman Chemical) in 1 ml of buffer (20 mM Tris-HCl, 0.2 M NaCl, 20 mM deoxycholate, pH 8.5) for 24 hours at room temperature [14]. The reaction mixture was extracted with one volume of methanol and 2 volumes of chloroform supplemented with 0.01% BHT. The chloroform layer was collected and dried under argon, and the oxidized AA-CE was reconstituted in hexane. Free radical oxidation of AA-CE was initiated by adding 20 mg of 2,2′-azobis (2,4-dimethylvaleronitrile) (AMVN; from Cayman Chemical) to 200 mg of AA-CE in 2.5 ml benzene and incubating the reaction mixture in an O_2_ atmosphere for 24 hours at 37°C [33]. After drying down the benzene solution under argon, the oxidized AA-CE was resuspended in hexane.

### Liquid Chromatography and Mass Spectrometry

Normal phase (NP) and reverse phase (RP) liquid chromatography (LC) was carried out using two Shimadzu LC-10AD high performance pumps interfaced with a Shimadzu SCL-10A controller. For analytical purposes, we used reverse phase LC with a 2.1 mm×250 mmVydac C18 column (catalog # 201TP52) equipped with a guard column held at 35°C. Buffer A was water/tetrahydrofuran (40/60, v/v) containing 5 mM ammonium acetate; buffer B was tetrahydrofuran. Gradient elution was achieved using 100/0 A/B at 0 min and linearly ramped to 50/50 A/B by 15 min. A/B was linearly ramped back to 100/0 by 17 min and held there until 25 min to achieve column re-equilibration. Mass spectral analyses were performed using an Applied Biosystems 4000 QTrap hybrid quadrupole linear ion trap mass spectrometer equipped with a Turbo V ion source, operating the ion source in positive electrospray, multiple reaction monitoring (MRM) mode. MRM pairs employed in the detection method used the ammoniated CE precursor mass and the cholesterol product fragment with m/z = 369 (exact mass 369.4). The cholesterol fragment is common to all CEs, regardless of their parent mass or moiety [14].

For semi-preparative LC, we performed two rounds of normal phase LC separation using 10 mm×250 mm Phenomenex silica columns (catalog # 00G-4274-N0). First, the sample was run at a flow rate of 4 ml/min in isocratic buffer C [hexane/isopropanol/water (970/29/1, v/v)]. Fractions with retention times between 10–13 min, which contained a compound with the precursor/product mass of 755/369, were subjected to a second round of isocratic LC with buffer D [hexane/isopropanol/water (980/20/0.2, v/v)] at 4 ml/min. Collected fractions were dried under argon, resuspended in hexane and split for MS analysis and for biological assays. Following the identification of the mass of a compound with the highest biological activity, it was subjected to silver ion coordination ion spray mass spectrometry analysis as described [34]. Although the sensitivity of (CE+Ag)^+^ detection is lower than that of (CE+NH_4_)^+^ ions, the Ag^+^ coordination produces Hock fragments from a hydroperoxide-containing oxidized CE, useful for molecule identification [34].

### Immunoblot Analysis

Antibodies specific to p-SYK, p-ERK1/2, p-JNK, p-c-Jun, and GAPDH were purchased from Cell Signaling Technology. Antibodies specific to c-myc, flag and gfp were purchased from Santa Cruz Biotechnology, Sigma-Aldrich and Abcam, respectively. Cell lysates were subjected to gel electrophoresis and immunoblot as described [25].

### Cytokine ELISA

BMDM (0.1×10^6^) were plated overnight and then incubated with 10 µg/ml control AA-CE or BEP-CE for 6 hours. Supernatants were collected and centrifuged at 10,000 rpm for 5 min to remove floating cells. Levels of CXCL2 (MIP-2) were measured in ELISA using reagents from R&D Systems.

### Macrophage Lipid Accumulation

To detect intracellular accumulation of neutral lipid, cells were stained with Oil Red O and hematoxylin or, alternatively, with LipidTox Red (Invitrogen) and the nuclear stain Hoechst 33358 (Sigma) as described previously [22]. In some experiments, macrophages were incubated with a 10,000 Da dextran labeled with Alexa Fluor 488 (Invitrogen) and stained with Alexa Fluor 568-phallodin (Invitrogen) to visualize F-actin. Bright field images were captured with NanoZoomer 2.0-HT (Hamamatsu) and fluorescent images were captured with a Delta Vision Digital Imaging System (Applied Precision). The images were deconvolved and volume views were generated by combining areas of maximal intensity of each optical section with SoftWorx programs.

### Statistical Analyses

Graphs represent means ± standard error from 3–4 independent experiments. Results were analyzed using Student’s t-test or one-way ANOVA and the differences with p<0.05 were considered statistically significant.

## Results

### BEP-CE is an Active Component of mmLDL

**Figure 1:**
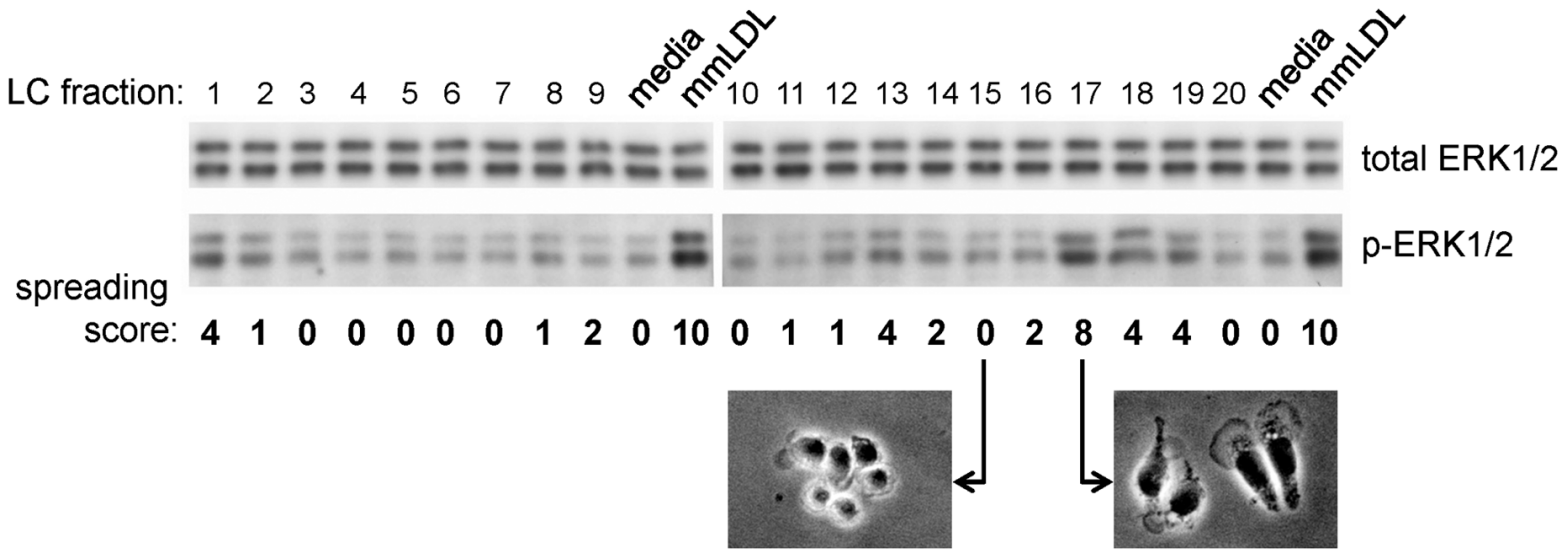
Screening for biologically active fractions isolated from 15LO-oxidized AA-CE. The product of 15LO-mediated oxidation of AA-CE was separated using one-step normal phase LC as described in Methods. The collected fractions were added to J774 macrophages for 15 min, and cell spreading was scored by two independent observers, with selected samples photographed in phase contrast. Cell lysates were analyzed for p-ERK1/2 and total ERK1/2 in immunoblot. Cells treated with media only and with mmLDL were used as negative and positive controls.

In a previous study, we identified OxCEs as components of mmLDL responsible for activation of macrophage inflammatory responses [14]. We further attempted to identify a specific, biologically active OxCE molecule, but the amount of OxCE material isolated from mmLDL proved to be insufficient for the detailed LC/MS analysis and biological assays. Because mmLDL is produced by incubating native LDL with 15LO-expressing cells, we then used OxCE derived from a reaction of AA-CE with 15LO. The product of this reaction induced biological responses in macrophages similar to those induced by mmLDL [14]. We therefore oxidized AA-CE in a 15LO enzymatic reaction and separated the resulting mixture of OxCE products by the LC technique described in Methods. The LC fractions were tested with J774 macrophages to assay their effects on cell spreading and phosphorylation of ERK1/2, the two robust effects of non-fractionated AA-CE/15LO and mmLDL [14]. As shown in Fig. 1, fraction #17 induced the most visible cell spreading and ERK1/2 phosphorylation. The dominant mass in #17 OxCE fraction had an m/z = 755 (ammonium adduct; exact mass 754.9 precursor and 369.4 product).

**Figure 2:**
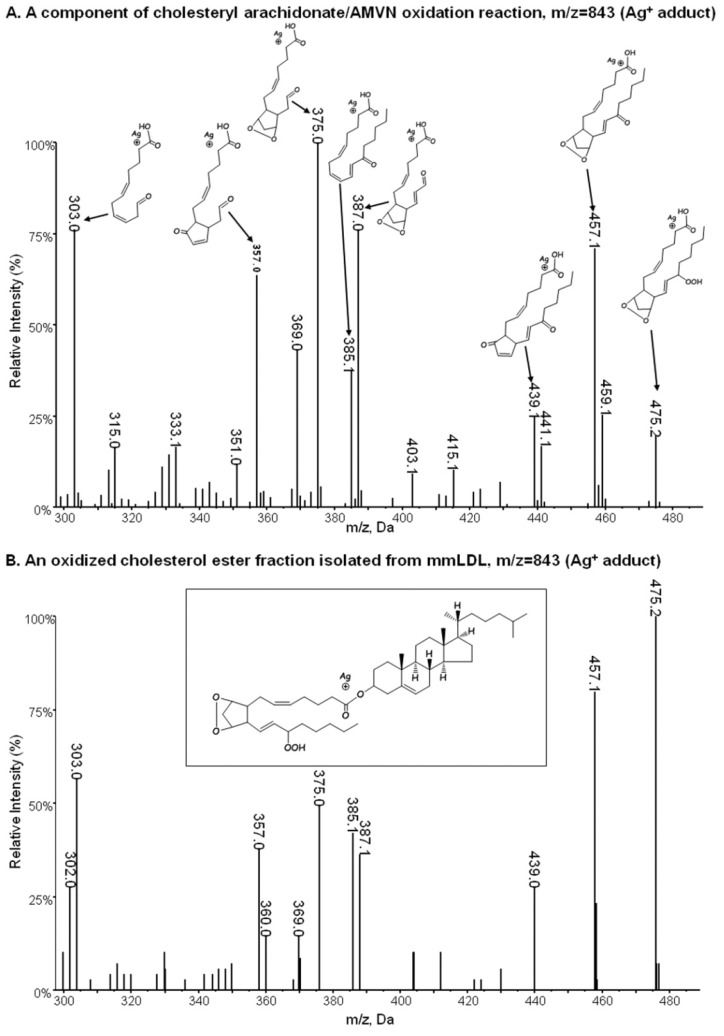
Fragmentation pattern of BEP-CE. AA-CE oxidized with AMVN and mmLDL lipids extract were subjected to two-step normal phase LC separation as described in Methods. Fractions collected between 7 and 8 min retention time from an LC column were subjected to silver ion coordination ion spray mass spectrometry analysis.

Based on a study from Porter’s group, who identified numerous products of free radical oxidation of AA-CE and of 15(S)-HETE [33], the m/z = 755 mass most likely represents an oxidized AA-CE with both bicyclic endoperoxide and hydroperoxide groups, here abbreviated as BEP-CE. Next, using the Porter group’s methodology, we reproduced their AMVN-initiated free radical oxidation of AA-CE and their separation protocol [33] to isolate the m/z = 755 compound and subjected it to Ag^+^ CIS-MS/MS as they described [34]. The fragmentation pattern of an m/z = 755 AA-CE/AMVN product was consistent with the structure of cholesteryl (9,11)-epidioxy-15-hydroperoxy-(5*Z*,13*E*)-prostadienoate (Fig. 2A), or BEP-CE, although the exact stereochemistry of the product was not determined in our study. Remarkably, the m/z = 755 OxCE fraction isolated from mmLDL had a very similar fragmentation pattern (Fig. 2B), suggesting the presence of BEP-CE in mmLDL. Because the AA-CE/AMVN reaction (see Methods) produces BEP-CE with high yield, enabling its separation from the reaction mixture in quantities sufficient for biological assays, we used this method to produce and isolate BEP-CE for further experiments.

### The Importance of the Hydroperoxide Group for BEP-CE Biological Activity

**Figure 3:**
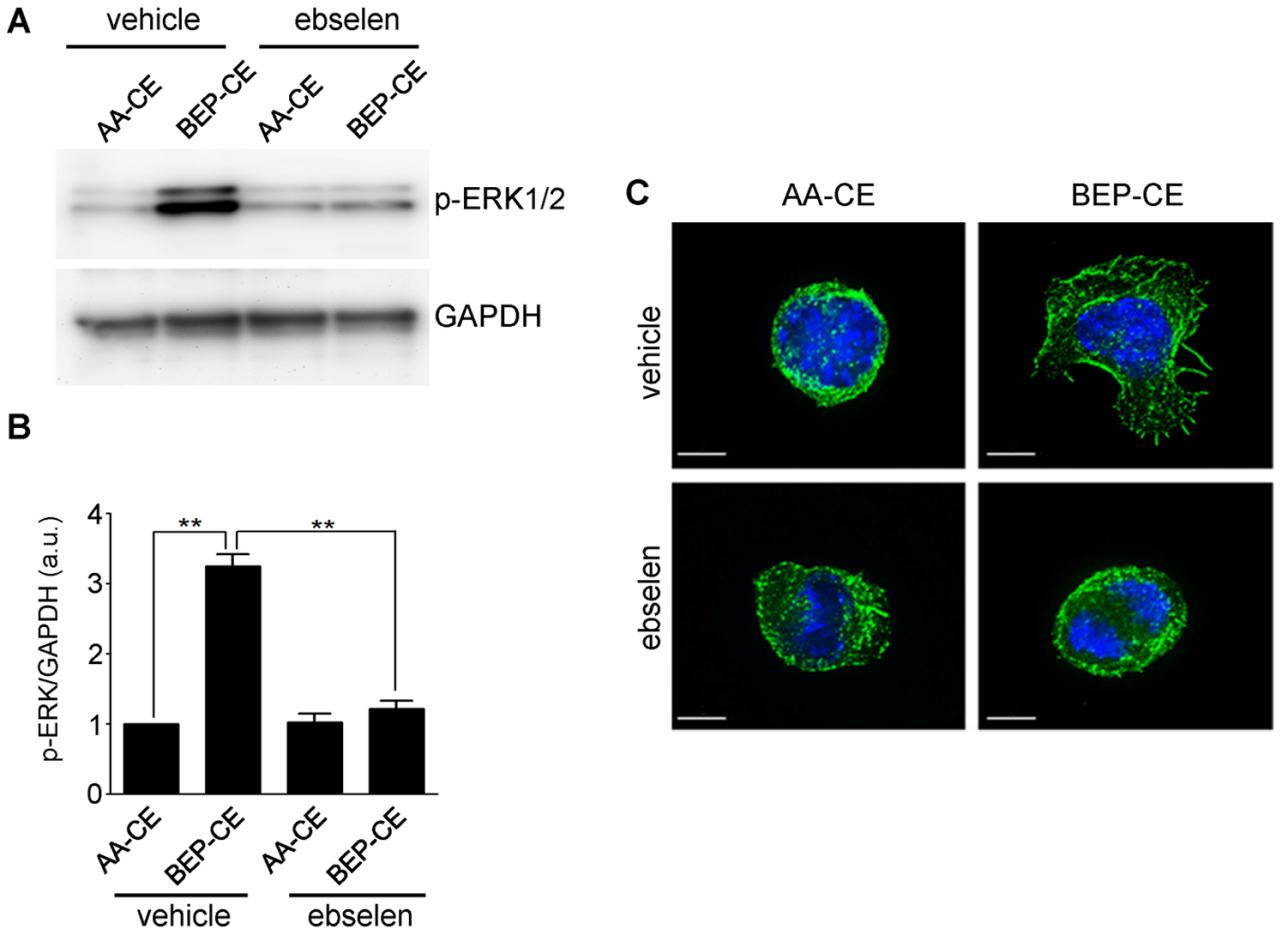
Ebselen inhibits BEP-CE-induced macrophage activation. Stock solutions (5 mg/ml) of AA-CE and BEP-CE were incubated with vehicle (DMSO) or 50 µM ebselen for 30 min. The AA-CE and BEP-CE were then added at 10 µg/ml to J774 macrophages and incubated for 15 min. **A,** p-ERK1/2 and GAPDH were detected by immunoblot analysis. **B,** ERK1/2 phosphorylation was quantified and normalized to GAPDH. Mean±SE; n = 3. **, p<0.005. **C,** Cells were stained with FITC-phalloidin and Hoechst 33358 to visualize F-actin (green) and the nuclei (blue). Scale bar, 5 µm.

We previously reported that ebselen, which specifically reduces hydroperoxides, diminished mmLDL-induced cell spreading and phosphorylation of ERK1/2 [14]. Thus, to examine whether the hydroperoxide group in BEP-CE is important for its biological activity, we pre-incubated AA-CE and BEP-CE with ebselen and then added the CEs to macrophages. As shown in Fig. 3, ebselen reduced BEP-CE-induced ERK1/2 phosphorylation and macrophage spreading, suggesting that the hydroperoxide group is essential for the BEP-CE activity.

### TLR4 and SYK Mediate BEP-CE Activation of Macrophages

**Figure 4:**
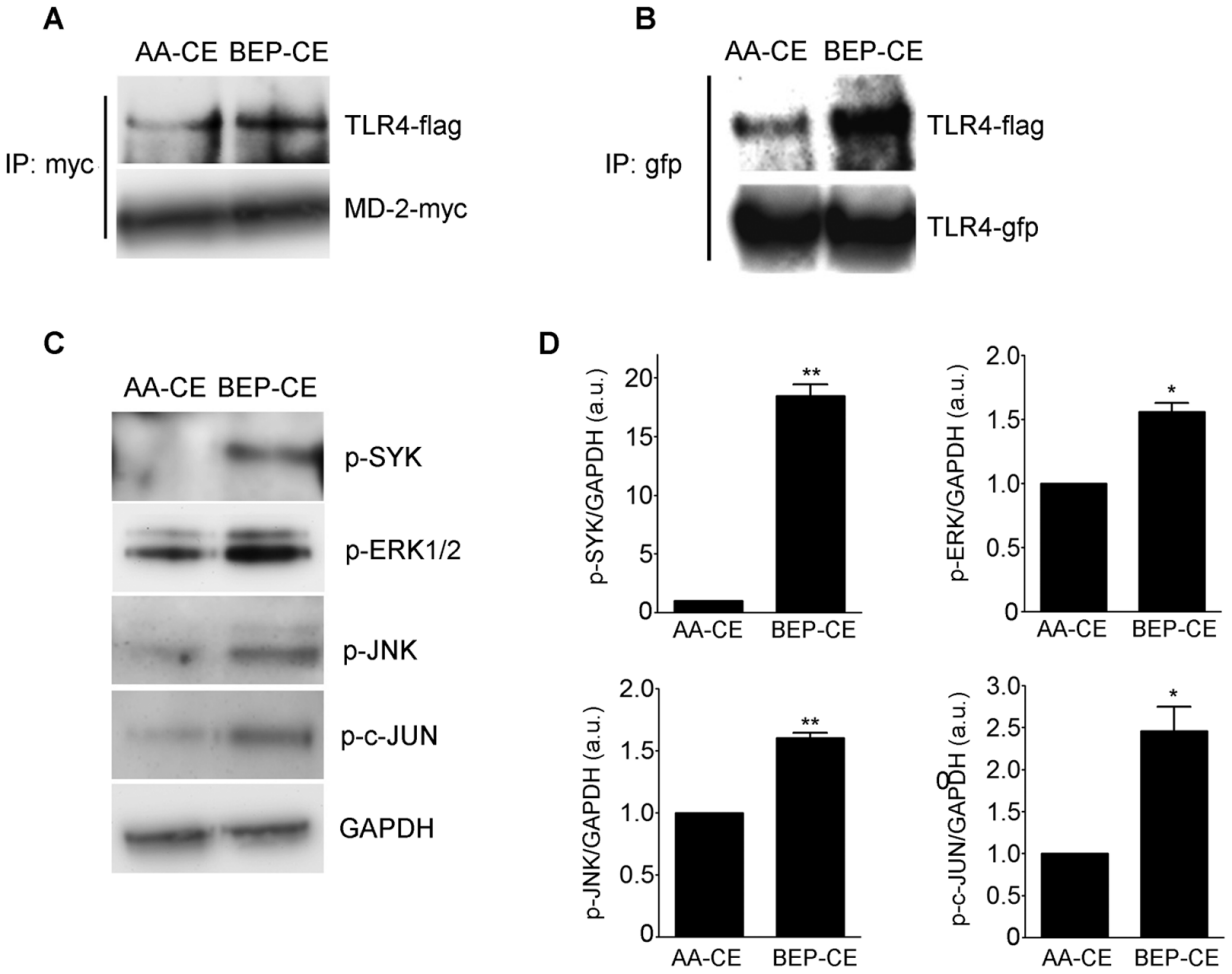
BEP-CE induces TLR4 dimerization and signaling in macrophages. **A**, Recombinant MD-2-myc-his and the extracellular domain of TLR4-flag-his were incubated with 10 µg/ml AA-CE or BEP-CE for 30 min at 37°C, followed by immunoprecipitation with an anti-myc antibody and immunoblot with anti-flag and anti-myc antibodies. **B,** Ba/F3 cells stably expressing TLR4-flag and TLR4-gfp were incubated with 10 µg/ml AA-CE or BEP-CE for 30 min. Cell lysates were immunoprecipitated with an anti-gfp antibody and immunoblotted with anti-flag and anti-gfp antibodies. **C,** J774 cells were incubated with 10 µg/ml AA-CE or BEP-CE for 15 min and cell lysates were immunoblotted with indicated antibodies. **D,** Quantification of protein phosphorylation, normalized to GAPDH. Mean±SE; n = 3. *, p<0.05; **, p<0.005.

**Figure 5:**
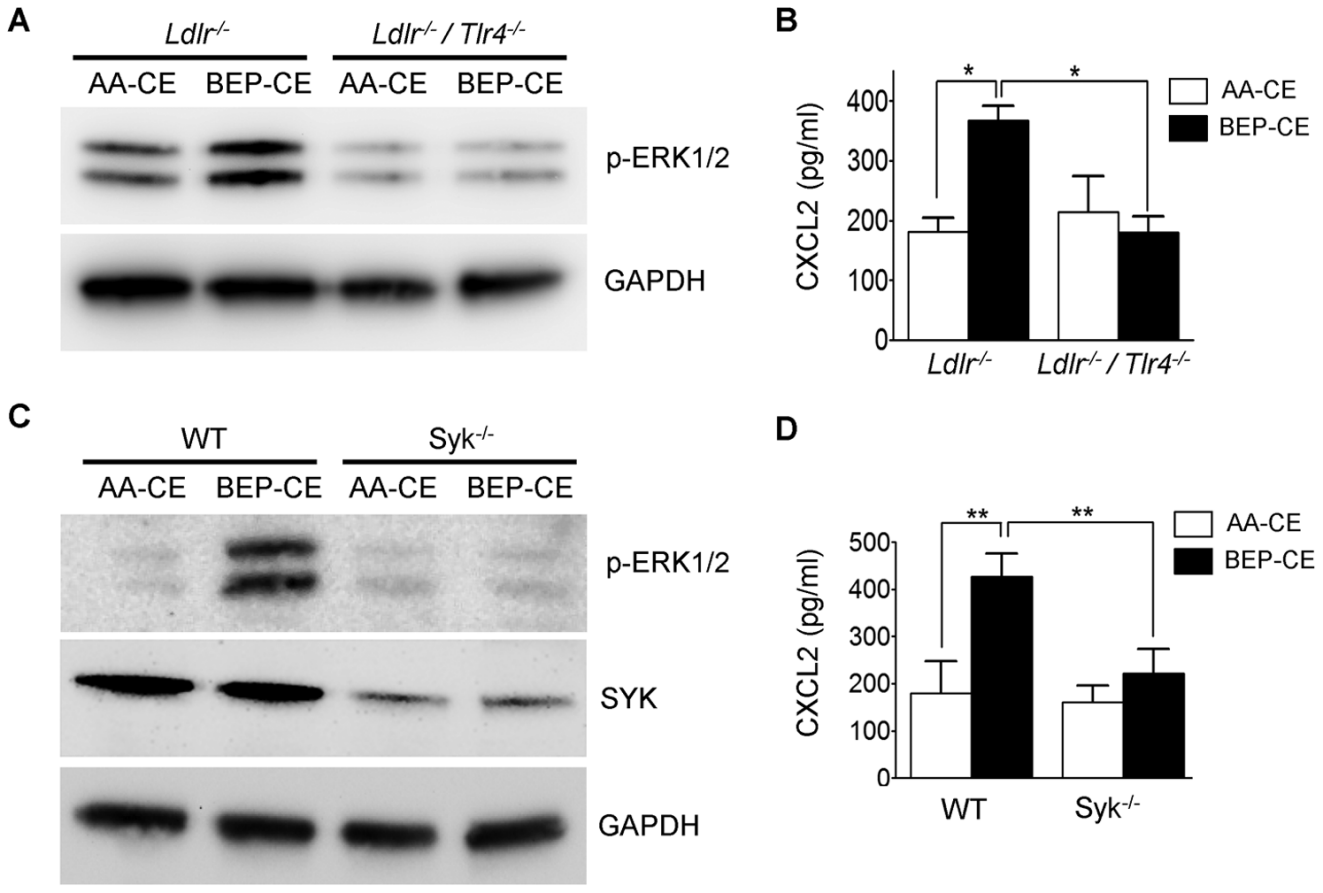
TLR4 and SYK deficiency inhibit macrophage response to BEP-CE. BMDM isolated from *Ldlr^−/−^* or *Ldlr^−/−/^Tlr4^−/−^* (**A** and **B**) and WT or *Syk^−/−^* (**C** and **D**) mice were incubated with 10 µg/ml AA-CE or BEP-CE for 15 min to detect ERK1/2 phosphorylation (**A** and **C**) or for 6 hours to measure secreted CXCL2 (**B** and **D**). SYK knockdown in BMDM was confirmed in the blot shown in panel **C**. Mean±SE; n = 3–4. *, p<0.05; **, p<0.005.

In previous studies, we demonstrated the importance of a TLR4/SYK signaling pathway in mediating mmLDL-induced inflammatory responses in macrophages [22], [23], [25]. In the current study, we asked whether BEP-CE also activated the TLR4/SYK signaling cascade. First, we tested initial steps in the pathway, MD-2/TLR4 and TLR4/TLR4 dimerization. For these studies, we generated HEK293 cell lines stably expressing and secreting MD-2 and the extracellular domain of TLR4. The recombinant proteins were incubated with AA-CE or BEP-CE and were subjected to MD-2 pull down and TLR4 and MD-2 immunoblotting. The data shown in Fig. 4A indicates that BEP-CE induces MD-2/TLR4 binding. Next, we used Ba/F3 cells stably expressing cell-associated TLR4-gfp and TLR4-flag [29], [30]. The pull-down/immunoblot assay shown in Fig. 4B demonstrates that BEP-CE induces TLR4 dimerization. To evaluate TLR4 downstream signaling events specific for the mmLDL macrophage activation [35], we examined phosphorylation of SYK, ERK1/2, JNK, and c-Jun. These signaling proteins were phosphorylated in macrophages stimulated with BEP-CE (Fig. 4C and D). To assess the requirement for TLR4 and SYK in BEP-CE-induced activation of primary macrophages, we used BMDM isolated from wild type and knockout mice. While BEP-CE induced ERK1/2 phosphorylation and CXCL2 secretion by WT macrophages, both TLR4- and SYK-deficient BMDM did not respond to BEP-CE (Fig. 5).

### BEP-CE Induces Foam Cell Formation

**Figure 6:**
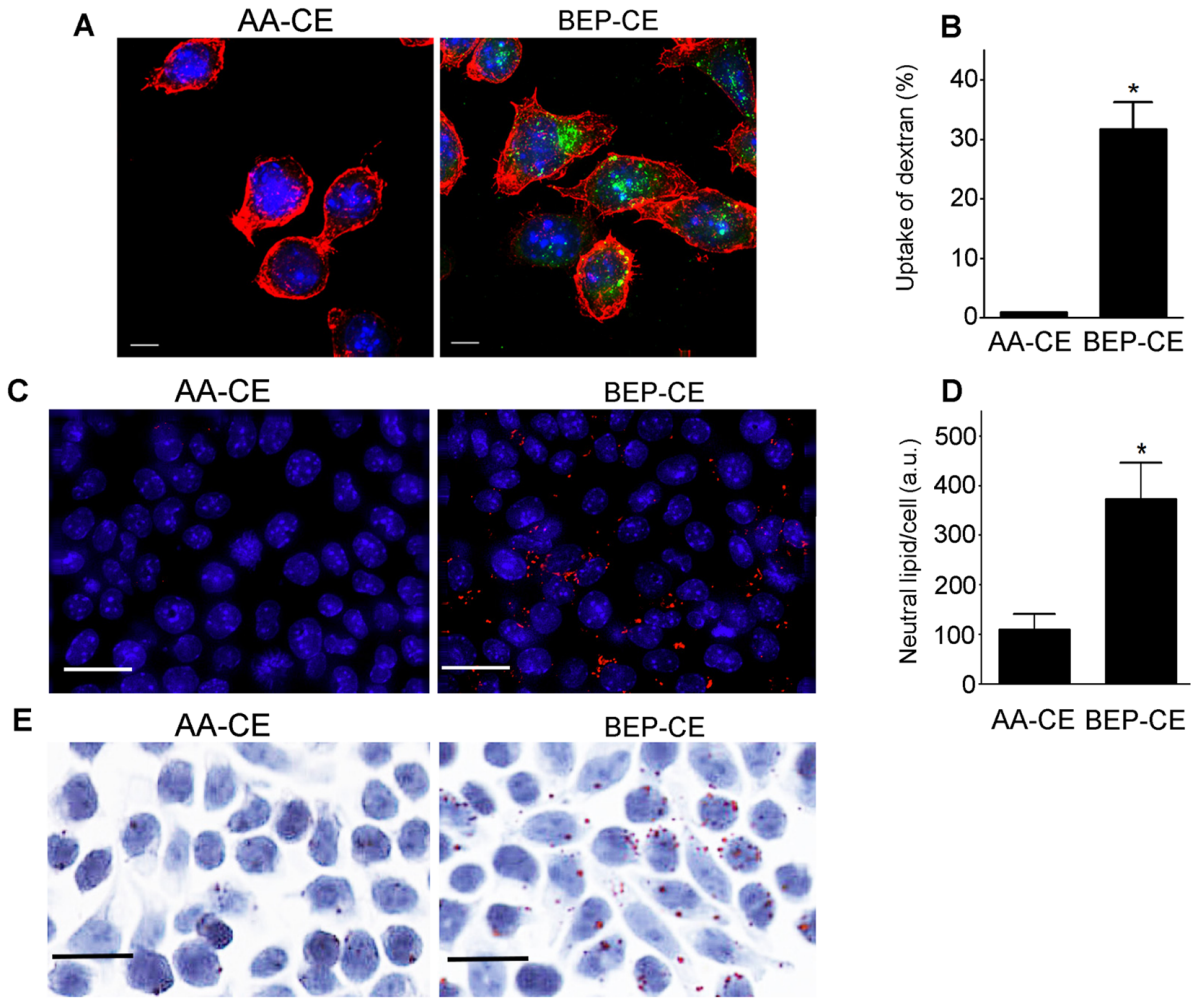
BEP-CE induces macropinocytosis and lipid accumulation in macrophages. **A** and **B,** J774 cells were incubated with Alexa Fluor 488-labeled dextran (10,000 Da), in the presence of 10 µg/ml AA-CE or BEP-CE, for 30 min. Cells were stained for F-actin (red) and nuclei (blue) and imaged (**A,** scale bar, 10 µm) or analyzed by flow cytometry to quantify the number of cells that internalized fluorescent dextran (**B**). **C**–**E**, J774 cells were incubated with 200 µg/ml native LDL (protected from oxidation with BHT), in the presence of 10 µg/ml AA-CE or BEP-CE, for 40 hours and stained for neutral lipid with LipidTox (red) and nuclei (blue) (**C**), or with Oil Red O and counterstained with hematoxilin (**E**). Scale bar, 20 µm. Lipid deposits were quantified by measuring the area of LipidTox staining per cell (**D**). A total of 73 and 111 cells in AA-CE and BEP-CE samples, respectively, were measured in 3 independent experiments. Mean±SE. *, p<0.05.

Lipid-laden macrophage foam cells play a key role in the development of atherosclerosis. We previously demonstrated that mmLDL induces macropinocytosis, resulting in lipoprotein uptake by macrophages [22]. In this study, we demonstrated that BEP-CE also induced macropinocytosis as detected by macrophage uptake of fluorescent dextran (Fig. 6 A and B). As with mmLDL, the BEP-CE-induced macropinocytosis resulted in uptake of native LDL and macrophage lipid accumulation as detected with the fluorescent stain for neutral lipid LipidTox Red and with Oil Red O (Fig. 6 C–E).

### BEP-CE in Human Plasma and Atherosclerotic Lesions

**Figure 7:**
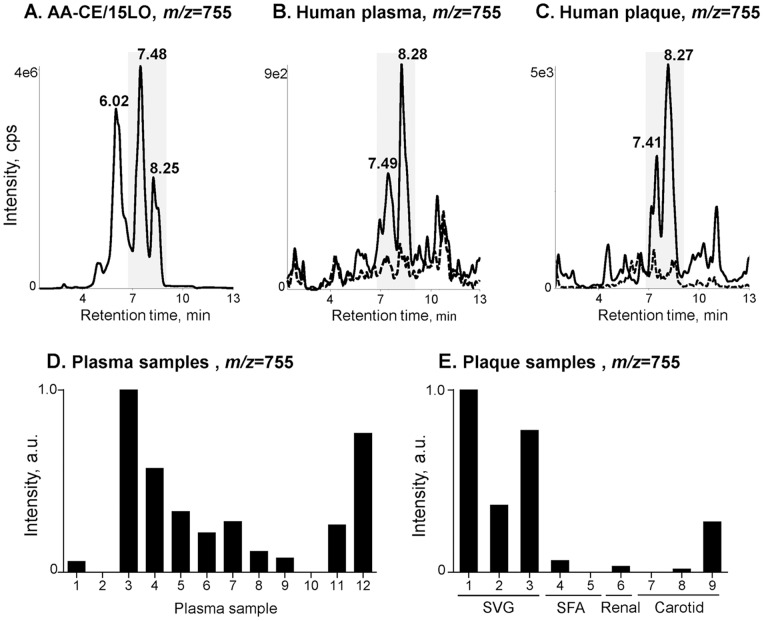
BEP-CE in human plasma and atherosclerotic lesions. LC-MS/MS spectra obtained with the MRM method detecting the 754.9 precursor and 369.4 product masses. **A,** Product of an AA-CE/15LO reaction. **B,** Lipid extracts from plasma samples obtained from patients presented for cardiac catheterization. Shown are 2 out of 12 samples tested, corresponding to # 3 (solid line) and #8 (dashed line) in panel **D**. **C,** Lipid extracts from the embolic material captured in distal protection devices during saphenous vein graft (solid line; #1 in **E**) and carotid artery (dashed line; #8 in **E**) interventions. Samples in panels **B** and **C** were collected from different patients. Shaded are the peaks common for all three groups of samples. **D,** Relative intensity (arbitrary units) of the *m/z* = 755, 8.28 min retention time signal in 12 human plasma samples tested. **E,** Relative intensity (arbitrary units) of the *m/z* = 755, 8.27 min retention time signal in human plaque samples. SVG, saphenous vein graft; SFA, superficial femoral artery; Renal, renal artery; Carotid, carotid artery.

We have previously documented the presence of OxCE and specifically an OxCE with m/z = 755 in murine atherosclerotic lesions as well as in zebrafish fed a high cholesterol diet [8], [14]. To determine the relevance of this for humans, in the current study, we used a similar LC-MS/MS technique to examine for BEP-CE in human samples. We found a CE with m/z = 755 and eluting at retention times, similar to those of the BEP-CE product of the AA-CE/15LO reaction, in plasma of patients who presented for a clinically necessary cardiac catheterization, as well as in the plaque material released from a ruptured atheroma and captured in distal protection devices during percutaneous coronary and peripheral artery interventions (shaded peaks in Fig. 7A–C). Graphs in Fig. 7D and E show relative amounts of BEP-CE in 12 plasma and 9 plaque samples tested.

## Discussion

We identified BEP-CE, a polyoxygenated cholesterol ester with m/z = 755 and a fragmentation pattern consistent with a product of cholesteryl arachidonate oxidation with bicyclic endoperoxide and hydroperoxide groups, as an OxCE moiety that activates macrophages in a TLR4- and SYK-dependent manner. BEP-CE can be derived from AA-CE by free radical oxidation or enzymatic oxidation with 15LO, and it is a major biologically active fraction of mmLDL. BEP-CE induces TLR4 dimerization, activates SYK, ERK1/2, JNK and c-Jun, and induces cell spreading and lipid accumulation by macrophages, the biological effects characteristic of mmLDL. However, unlike mmLDL, BEP-CE induces only minimal activation of Akt (not shown), underscoring the complexity of mmLDL as a model of early stages of LDL oxidation occurring *in vivo*. In addition to OxCE, mmLDL may contain other oxidized lipids and serve as a carrier of other, non-lipid, biologically active molecules. BEP-CE is present in murine atherosclerotic lesions and in zebrafish fed a high cholesterol diet. Importantly, we now show the presence of BEP-CE in human plasma and human atherosclerotic lesion samples. Studies to quantify BEP-CE in a large set of clinical samples and to develop techniques to readily measure BEP-CE for biomarker assays are ongoing.

The mouse 12/15LO enzyme has been suggested as a major contributor to *in vivo* LDL oxidation during the development of diet-induced atherosclerosis. The 12/15LO knockout *Apoe* ^−/−^ mice fed an HFD have less atherosclerosis, lower titers of autoantibodies against OxLDL in plasma and lower isoprostane levels in urine as compared to *Apoe* ^−/−^ mice [36], [37]. Other studies have also established the importance of 12/15LO in hypercholesterolemic murine models, including knockout and transgenic mice fed an HFD [36]–[43]. Evidence of the association of a human 15LO polymorphism with the risk of cardiovascular disease is mixed: 15LO gene variants have been reported to associate with carotid plaque formation (but not carotid intima-media thickness) [44], and integrative predictive models include *Alox15* (gene encoding human 15LO) polymorphism as a factor in the development of coronary artery calcification in atherosclerosis [45], as well as in enhanced expression of IL-6, TNFα and IL-1β [46]. In contrast, other groups, reporting different *Alox15* SNPs, found no association of 15LO polymorphism with myocardial infarction [47], [48]. Heterozygote carriers of a near null variant of 15LO had an increased risk of coronary artery disease, but homozygote carriers, although rare, were associated with a non-significantly decreased risk of coronary artery disease [49]. No studies have yet correlated 15LO polymorphisms with markers of *in vivo* LDL oxidation, which are needed to interpret such associations.

We and others have demonstrated the presence of 15LO in human lesions, which colocalizes with OxLDL [50], [51]. However, there is also controversy about the findings of 15LO-derived oxidation products in human lesions [16], [52]–[54]. A confounding factor in interpreting such human data is the complex nature of *in vivo* oxidation, where an oxidative process initiated by 15LO can be further propagated via non-enzymatic, free radical mechanisms, as well as by enzymatic transformation of the initial products [16], [53], or by OxCE hydrolysis and remodeling [53].

In general, esterified eicosanoids are significantly more stable than corresponding free fatty acids [33], [55], and the bicyclic endoperoxide group is preserved even following brief exposure to aqueous solvent, as under the LC conditions employed. The presence of fragments with m/z 303 and 385 in the CID experiment (Fig. 2) is indicative of the intact bicyclic endoperoxide moiety in these structures. Although the m/z = 755 can be ascribed to an ammonium adduct of either BEP-CE or the corresponding isoketal (isolevuglandin), formed in protic conditions [56], the latter would have quickly reacted with proteins, resulting in covalent adducts and, thus, would have been undetectable in lipid extracts. In support of this stability, we were able to identify BEP-CE-like molecules in mmLDL, in human plasma (likely associated with lipoproteins) and even in extracts of lipid-rich atherosclerotic lesions.

There is evidence for a role of TLR4 in the development of human atherosclerosis as well. The common loss-of-function TLR4 polymorphism is associated with a decreased risk of carotid and femoral artery atherosclerosis and cardiovascular cause of death and reduced risk of acute coronary events, independent of other coronary risk factors [57]–[59]. However, the TLR4 polymorphism is not associated with coronary artery stenosis, cerebral ischemia, or progression of atherosclerosis in patients with familial hypercholesterolemia [60]–[62]. Because these studies evaluate different clinical manifestations of atherosclerosis, the observed discrepancies are not necessarily contradictory. Other studies showing increased TLR4 expression in macrophages in symptomatic carotid atherosclerotic plaques [63] and increased TLR4 expression in circulating monocytes of patients with coronary atherosclerosis [64] and patients with acute coronary syndrome compared to stable angina [65], [66], also suggest that TLR4 is involved in vascular inflammation in humans.

Studies of atherogenesis in mice support a role for TLR4 in the development of diet-induced atherosclerosis as well. Lipid accumulation and foam cell formation in early lesions of *Tlr4^−/−/^Apoe^−/−^* mice were reduced by 70–80% compared to *Apoe^−/−^* controls [67]. A deficiency in TLR4 or MyD88 (adaptor molecule for many TLRs) attenuates the development of atherosclerosis in hyperlipidemic *Apoe^−/−^* and *Ldlr^−/−^* mice [68], [69]. Richards et al. found less atherosclerosis in HFD-fed *Trif^−/−/^Ldlr^−/−^* but not *Tlr3^−/−/^Ldlr^−/−^* mice [70]. Since TRIF is an adaptor molecule transducing signals only from TLR4 and TLR3, this study also suggests an atherogenic role of TLR4. A study by Owens et al. in which *Ldlr^−/−^* mice were fed a HFD and infused with angiotensin II to induce abdominal aortic aneurism, found that TLR4 and MyD88 deficiency inhibited both atherosclerosis and aneurism formation [27]. However, the authors found no role for TLR4-deficient bone marrow-derived cells in the development of atherosclerosis in this model [27]. In contrast, transplantation of bone marrow from *Tlr4^−/−^* mice into *Ldlr^−/−^* recipients, followed by feeding a high-cholesterol, low-fat diet, resulted in reduced atherosclerosis compared with mice transplanted with wild type bone marrow [71]. Overall, these results imply that endogenous ligands activate TLR4 in vascular cells, leading to proatherogenic effects. We suggest that BEP-CE, arising under hypercholesterolemic and pro-oxidative conditions during atherogenesis, is one of the endogenous TLR4 ligands.
